# Elevated Antigen-Presenting-Cell Signature Genes Predict Stemness and Metabolic Reprogramming States in Glioblastoma

**DOI:** 10.3390/ijms26157411

**Published:** 2025-08-01

**Authors:** Ji-Yong Sung, Kihwan Hwang

**Affiliations:** Department of Neurosurgery, Seoul National University Bundang Hospital, Seoul National University College of Medicine, Seongnam-si 13620, Republic of Korea

**Keywords:** antigen-presenting-cell signature genes, glioblastoma, RNA sequencing, stemness, metabolic reprogramming, inflammation

## Abstract

Glioblastoma (GBM) is a highly aggressive and heterogeneous brain tumor. Glioma stem-like cells (GSCs) play a central role in tumor progression, therapeutic resistance, and recurrence. Although immune cells are known to shape the GBM microenvironment, the impact of antigen-presenting-cell (APC) signature genes on tumor-intrinsic phenotypes remains underexplored. We analyzed both bulk- and single-cell RNA sequencing datasets of GBM to investigate the association between APC gene expression and tumor-cell states, including stemness and metabolic reprogramming. Signature scores were computed using curated gene sets related to APC activity, KEGG metabolic pathways, and cancer hallmark pathways. Protein–protein interaction (PPI) networks were constructed to examine the links between immune regulators and metabolic programs. The high expression of APC-related genes, such as HLA-DRA, CD74, CD80, CD86, and CIITA, was associated with lower stemness signatures and enhanced inflammatory signaling. These APC-high states (mean difference = –0.43, adjusted *p* < 0.001) also showed a shift in metabolic activity, with decreased oxidative phosphorylation and increased lipid and steroid metabolism. This pattern suggests coordinated changes in immune activity and metabolic status. Furthermore, TNF-α and other inflammatory markers were more highly expressed in the less stem-like tumor cells, indicating a possible role of inflammation in promoting differentiation. Our findings revealed that elevated APC gene signatures are associated with more differentiated and metabolically specialized GBM cell states. These transcriptional features may also reflect greater immunogenicity and inflammation sensitivity. The APC metabolic signature may serve as a useful biomarker to identify GBM subpopulations with reduced stemness and increased immune engagement, offering potential therapeutic implications.

## 1. Introduction

Glioblastoma (GBM) is the most aggressive and heterogeneous form of primary brain tumor in adults, characterized by marked cellular plasticity, metabolic reprogramming, and resistance to therapy. Among the diverse cellular constituents of the GBM microenvironment, glioma stem-like cells (GSCs) play a pivotal role in tumor maintenance, recurrence, and therapeutic resistance due to their self-renewal capacity and adaptive metabolic states. Understanding the regulatory mechanisms that govern stemness and metabolic plasticity in GBM is therefore critical for the development of targeted therapies [1].

Glioblastoma (GBM) is not only defined by its histological aggressiveness but also by the profound alterations in cellular metabolism and immune dynamics that it causes. A hallmark of GBM is its ability to undergo metabolic reprogramming, allowing tumor cells to flexibly shift between glycolysis and oxidative pathways in response to environmental cues. Recent studies have underscored the importance of lipid metabolism, including enhanced fatty acid synthesis and β-oxidation, in sustaining the energy demands and survival of glioma stem-like cells (GSCs). These metabolic adaptations are tightly coupled with resistance to therapy and promote cellular plasticity within the tumor ecosystem. Compounding these metabolic alterations, GBM exhibits a markedly “immune cold” phenotype characterized by low T cell infiltration, dysfunctional antigen presentation, and the predominance of immunosuppressive cell types such as tumor-associated macrophages and regulatory microglia. In addition to tumor-associated macrophages, resident microglia are key contributors to the immunosuppressive microenvironment in GBM, where they exhibit altered antigen-presenting capacity and secrete cytokines that support tumor growth and immune evasion.

GSCs actively shape this immunosuppressive landscape by secreting anti-inflammatory cytokines, downregulating MHC expression, and remodeling the extracellular matrix, thereby evading immune surveillance. Together, these features form a hostile environment for effective immune activation and therapy, positioning immune and metabolic escape as dual barriers to GBM eradication. In this context, understanding how antigen-presenting-cell (APC) gene signatures intersect with metabolic and stemness states offers a unique lens into the functional heterogeneity of GBM and may uncover therapeutic vulnerabilities linked to immune visibility and metabolic rigidity.

Recent studies have highlighted the influence of the immune microenvironment in shaping the phenotypic states of GBM cells. In particular, antigen-presenting cells (APCs)—including dendritic cells, macrophages, and microglia—play dual roles in initiating antitumor immunity and modulating local inflammatory responses. APCs express signature genes such as MHC class II molecules (e.g., *HLA-DRA* and *CD74*), costimulatory molecules (e.g., *CD80* and *CD86*), and regulatory transcription factors (e.g., *CIITA* and *IRF8*) that orchestrate T cell priming and antigen processing. Interestingly, these APC-associated signatures are increasingly recognized not only as markers of immune function but also as correlates of non-immune processes such as differentiation, cell fate determination, and metabolism in the tumor context [2,3,4].

Here, we investigated the hypothesis that elevated APC signature gene expression in GBM is associated with distinct cellular states, including reduced stemness and reprogrammed metabolic activity. By integrating transcriptomic data across both bulk- and single-cell levels, we demonstrate that APC signatures are enriched in more differentiated, inflammatory GBM states and are inversely correlated with GSC-associated programs. Furthermore, we identified specific metabolic pathways, such as glycolysis and lipid metabolism, that are co-regulated with APC gene expression, suggesting a functional link between immune gene activity and tumor cell-intrinsic metabolic shifts [5,6,7].

These findings position APC gene signatures as potential biomarkers for delineating functional heterogeneity within GBM and offer a novel perspective on how immune signaling intersects with core tumor-cell programs. Understanding this interplay may open new avenues for combining metabolic and immunologic therapies in GBM treatment.

### 1.1. Key Points

Elevated APC gene signatures in GBM define a less stem-like, more metabolically differentiated cellular state. This shift may indicate increased immunogenicity and a greater responsiveness to inflammatory signals.

### 1.2. Importance of the Study

In this study, we revealed a significant association between APC signature gene expression and cellular state dynamics in glioblastoma (GBM). Elevated APC signatures, including key immune regulatory genes such as *HLA-DRA, CD74, CD86,* and *CIITA*, correlate with reduced stemness, enhanced inflammatory signalling, and distinct metabolic remodelling. These APC-high states reflect a transition from glycolytic, stem-like phenotypes toward more differentiated, immunologically active, and metabolically specialized tumor cell populations.

Our findings challenge the conventional view that inflammation uniformly promotes stemness and tumor aggressiveness in GBM. Instead, they indicate that, in specific cellular contexts, pro-inflammatory signaling and antigen-presenting programs may drive stemness attenuation and metabolic differentiation. This insight carries important therapeutic implications: tumors exhibiting high APC gene expression may be more amenable to immunotherapies or metabolic inhibitors and less likely to undergo treatment-resistant recurrence mediated by glioma stem-like cells.

Overall, this work underscores the value of APC-related transcriptional signatures as integrative biomarkers reflecting tumor cell identity, metabolic state, and immune engagement.

## 2. Results

### 2.1. Prognosis-Related Genes Are Associated with APC Signature Genes

**Figure 1:**
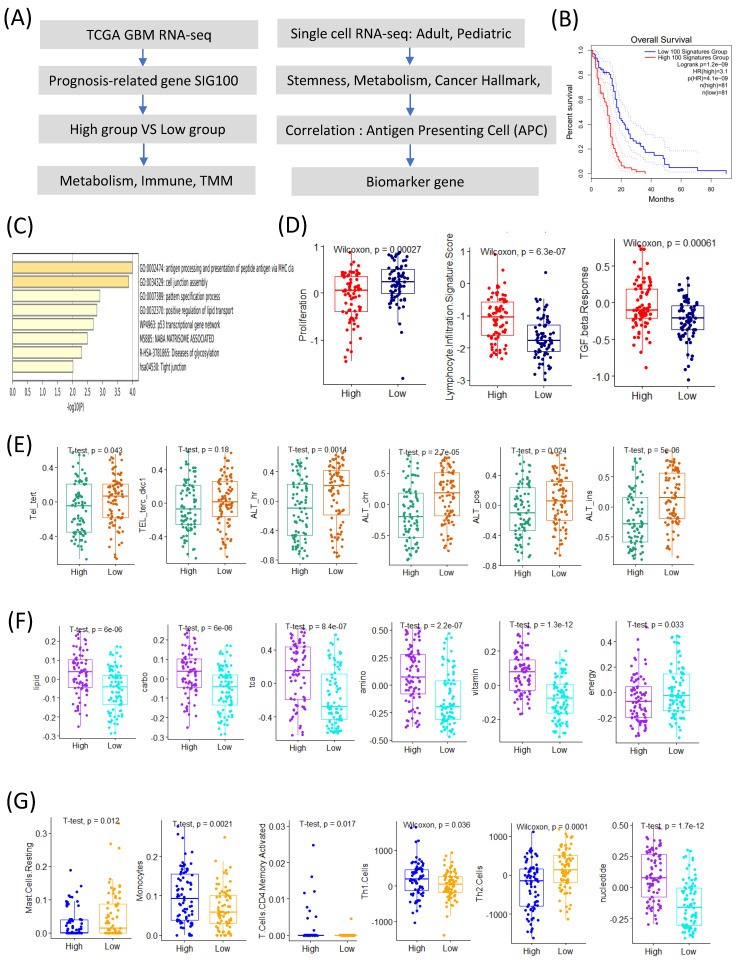
Prognosis-related genes are associated with antigen-presenting cell signature genes. (**A**) Schematic overview of the computational pipeline used in this study. (**B**) Kaplan–Meier survival curves comparing the SIG100-high and SIG100-low groups. (**C**–**G**) Correlation coefficients (Pearson’s r) and adjusted *p*-values (FDR of <0.05) are indicated. For example, SIG100 score and HLA-DRA expression: r = 0.61, FDR-adjusted *p* < 0.001. (**C**) Bar graph showing pathways significantly enriched in the high SIG100 group. (**D**) Box plots comparing proliferation, lymphocyte infiltration signature scores, and TGF-β response between the high and low SIG100 groups. (**E**) Box plots showing telomere maintenance mechanism activities (e.g., telomerase and alternative lengthening of telomeres) in the high versus low SIG100 groups. (**F**) Box plots comparing the activities of six metabolic pathways between the high and low SIG100 groups. (**G**) Box plots depicting the relative abundances of mast-cell resting, monocytes, activated CD4 memory T cells, Th1 cells, Th2 cells, and nucleotides in the high and low SIG100 groups.

We identified the top 100 prognosis-related signature genes using the TCGA GBM transcriptome data. Based on the signature scores calculated from these genes, patients with GBM were stratified into high- and low-score groups. Patients in the high-score group exhibited significantly poorer overall survival compared to those in the low-score group (Figure 1A,B).

A gene ontology (GO) enrichment analysis revealed that the genes highly expressed in the high-score group were significantly enriched in the pathways related to antigen processing and the presentation of peptides via MHC class I, the positive regulation of lipid transport, the p53 transcriptional gene network, and NABA matrisome-associated processes (Figure 1C). Conversely, the low-score group showed a significantly higher proliferation signature (Figure 1D).

The immune-related analyses demonstrated that the high-score group was associated with elevated lymphocyte infiltration and TGF-β response signatures (Figure 1D). Interestingly, among the telomere maintenance mechanisms (TMMs), the alternative lengthening of telomeres (ALT) signature was significantly higher in the low-score group compared to its telomerase-related activity (Figure 1E).

Among the seven major metabolic pathways, six (lipid, carbohydrate, TCA cycle, amino acid, vitamin, and nucleotide metabolism) [8] were upregulated in the high-score group, whereas energy metabolism was significantly elevated in the low-score group (Figure 1F).

The immune-cell profiling revealed distinct differences between the groups. The low-score group exhibited significantly higher scores for resting mast-cells and Th2 cell signatures, whereas the high-score group showed increased activity for monocytes, activated CD4 memory T cells, and Th1 cells (Figure 1G) [9,10].

Collectively, these results suggest that the 100 prognosis-related signature genes are closely linked to APC characteristics and provide insights into the tumor microenvironmental landscape of patients with GBM with different prognoses.

### 2.2. At the Single-Cell Level, TMM Expression Varies Depending on the Degree of Stemness

**Figure 2:**
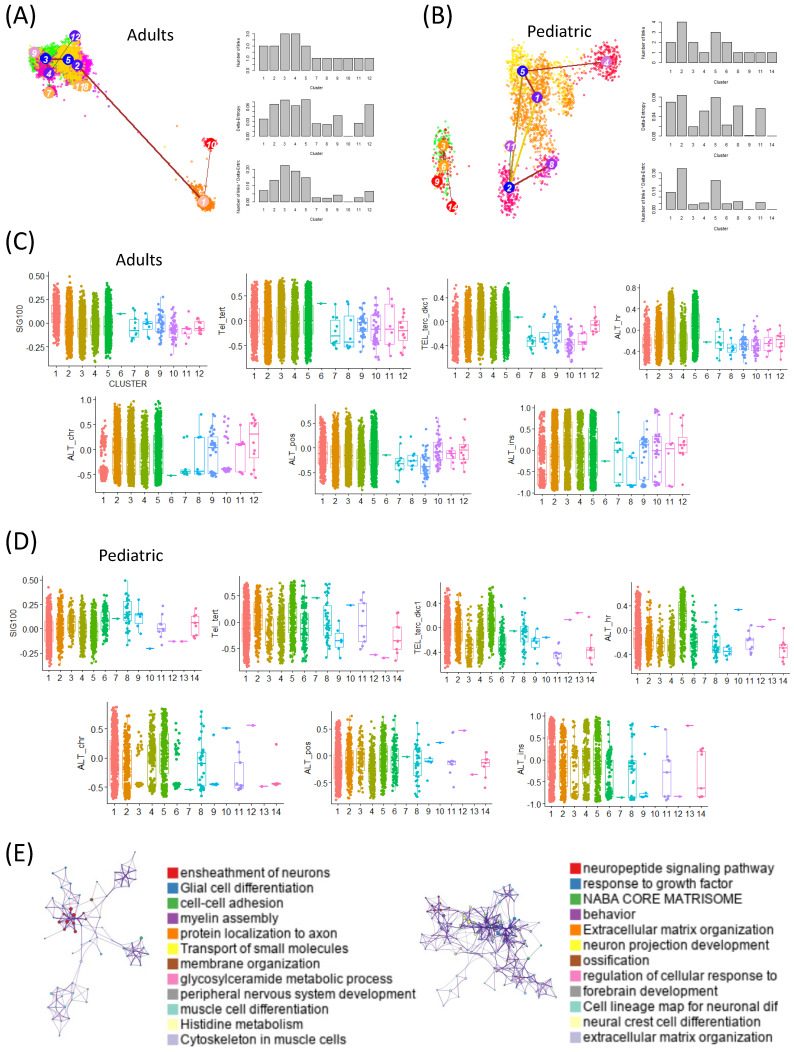
TMM expression varies with stemness at the single-cell level. (**A**) t-SNE plots illustrating the distributions of T cells, macrophages, tumor cells, and oligodendrocytes, with stemness levels inferred using the StemID algorithm for the adult GBM dataset. UMAP-based clustering of tumor cells from adult and pediatric samples, highlighting stemness-enriched populations. Dimensionality reduction and unsupervised clustering were performed on single-cell transcriptomic data from adult (left) and pediatric (right) tumor samples. Each point represents a single cell, colored by cluster identity (numerical labels indicate cluster numbers). Notably, clusters highlighted in royal blue correspond to populations with the highest stemness scores, indicating elevated self-renewal and plasticity. These royal-blue clusters are positioned centrally in the adult cohort and more dispersed yet interconnected in the pediatric group, suggesting distinct topological organizations of stem-like cell populations between age groups. (**B**) t-SNE plots showing the same cell types and inferred stemness levels for the pediatric GBM dataset. (**C**) Box plots comparing the SIG100 and telomere maintenance mechanism (TMM) activity across the StemID clusters for the adult samples. (**D**) Box plots of the SIG100 and TMM activity across the StemID clusters for the pediatric samples. (**E**) Gene-ontology network diagrams of the pathways enriched in the high-entropy cells for the adult (left) and pediatric (right) datasets.

We analyzed stemness at the single-cell level in both adult and pediatric GBM patients using scRNA-seq datasets and the StemID tool. Cells with high entropy were interpreted as having high stemness. In the adult dataset, clusters 3 and 5 were identified as high-entropy clusters, whereas in the pediatric dataset, clusters 5 and 2 showed high entropy (Figure 2A,B).

Clusters with high entropy were further validated to exhibit the elevated expression of canonical glioma stem-like cell markers (SOX2, OLIG2, PROM1), supporting their classification as high-stemness populations beyond theoretical entropy alone.

In the adults, we assessed the activity of a prognosis-associated gene signature (SIG100) across the clusters and observed significantly higher activity in the low-entropy clusters (clusters 1 and 10). Conversely, TMM activity was elevated in the high-entropy clusters. A similar pattern was observed in the pediatric dataset, where SIG100 activity was enriched in the low-entropy clusters while TMM activity was higher in the high-entropy clusters (Figure 2C,D).

To further investigate these patterns, we performed a gene-ontology (GO) enrichment analysis on the differentially expressed genes in the high- and low-entropy cells. In adults, the high-entropy cells were enriched for pathways related to the ensheathment of neurons, glial cell differentiation, cell–cell adhesion, myelin assembly, and protein localization in axons. In contrast, the pediatric high-entropy cells were enriched for the neuropeptide signaling pathway, responses to growth factor, NABA core matrisome, and extracellular matrix organization (Figure 2E).

These results indicate that stemness at the single-cell level is closely associated with both the SIG100 signature and TMM activity, with variations between adult and pediatric GBM patients. Importantly, this analysis revealed differentiation trajectories and transition patterns related to stemness that were not detectable in the bulk RNA-seq analyses.

### 2.3. Metabolic Reprogramming and Cancer Hallmark Pathways Are Heterogeneous Depending on the Activity of APC Signature Genes

**Figure 3:**
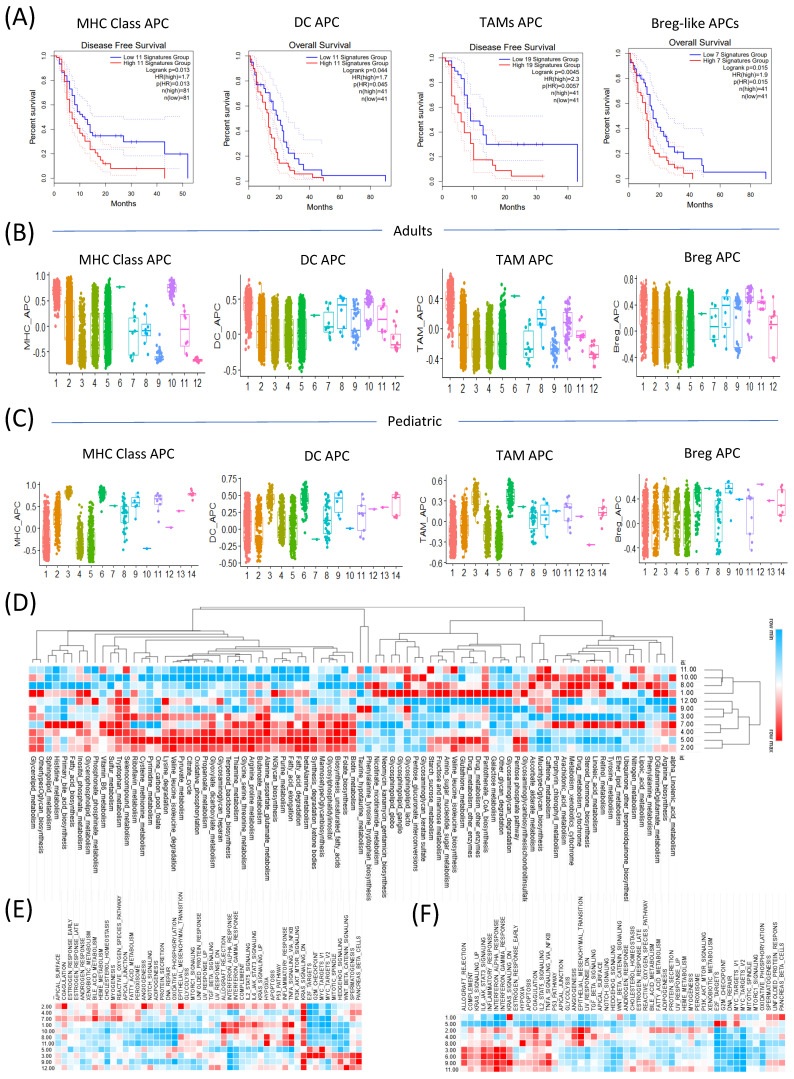
Metabolic reprogramming and cancer hallmark pathways vary with antigen-presenting-cell (APC) signature activity. (**A**) Kaplan–Meier plots showing the overall survival rates for the high- and low-MHC class APC, dendritic cell (DC) APC, tumor-associated macrophage (TAM) APC, and Breg-like APC expression groups. (**B**) Box plots of the MHC class APC, DC APC, TAM APC, and Breg-like APC activity in the StemID cluster of the adults. (**C**) Box plots of the MHC class APC, DC APC, TAM APC, and Breg-like APC activity in the StemID cluster of the pediatric dataset. (**D**) Heatmap of metabolic reprogramming in the StemID cluster for the adult dataset. “Heatmaps show Pearson correlation coefficients (r) between APC gene expression and metabolic pathway activity across single-cell clusters. Only correlations with FDR-adjusted *p*-values < 0.05 (Benjamini–Hochberg correction) are shown.” (**E**) A heatmap of the cancer hallmark pathways in the StemID cluster for the adult dataset. (**F**) A heatmap of the cancer hallmark pathways in the StemID cluster for the pediatric dataset.

We first investigated the prognostic value of APC-related signature genes in the TCGA GBM data. Across all four APC-related categories (MHC class APCs, dendritic cell (DC) APCs, tumor-associated macrophage (TAM) APCs, and Breg-like APCs), patients with high expression levels consistently exhibited poorer overall survival (MHC class APCs: *p* = 0.013; DC APCs: *p* = 0.044; TAM APCs: *p* = 0.0045; and Breg-like APCs: *p* = 0.015) (Figure 3A).

To better understand the cellular basis of these APC signatures, we analyzed the single-cell RNA-seq data to determine how APC-related gene expression varied across the clusters defined by stemness levels. Notably, APC gene activity was enriched in the low-entropy, low-stemness clusters, whereas the high-entropy, high-stemness clusters exhibited markedly reduced APC gene expression. This trend was consistently observed in both the adult and pediatric GBM datasets, suggesting a conserved relationship between stemness status and antigen presentation capacity across age groups (Figure 3B,C).

These observations support the notion that increased stemness is associated with immune evasion, potentially via the suppression of APC-related pathways. This aligns with cancer hallmarks such as immune escape and metabolic reprogramming.

To further explore this link, we examined the changes in metabolic pathway activity corresponding to stemness states at the single-cell level in the adult GBM patients. Distinct metabolic profiles were observed between the clusters as follows: cluster 1 (low entropy, low stemness) showed elevated activity of glycosphingolipid metabolism, whereas cluster 5 (high entropy, high stemness) exhibited increased activation of the glycosaminoglycan metabolism and oxidative phosphorylation (OXPHOS) pathways (Figure 3D). These findings suggest that stemness-driven metabolic reprogramming may contribute to the immunosuppressive microenvironment in GBM.

We further analyzed the activity of cancer hallmark pathways across the clusters in both the adult and pediatric GBM patients. In the adult dataset, the low-entropy, low-stemness clusters exhibited increased activity of the immune-related pathways such as allograft rejection, interferon-α response, and interferon-γ response. Notably, inflammatory response and TNF-α signaling via NF-κB were also more active in these low-stemness clusters, indicating a robust pro-inflammatory immune environment (Figure 3E).

In the pediatric dataset, the separation between clusters based on stemness was even more pronounced. Clusters 3, 6, and 9, characterized by low stemness, showed enriched activity of the inflammatory response pathways, whereas cluster 5, defined by high stemness, exhibited elevated activity in the proliferative and cell cycle-related pathways, including E2F targets, G2M checkpoint, and MYC targets V1 (Figure 3F).

These findings suggest that the metabolic reprogramming observed in the high-stemness clusters was associated with features of the immunosuppressive microenvironment in GBM, rather than implying a direct causal relationship. Furthermore, the distinct metabolic and immune circuit activities observed at the single-cell level highlighted the heterogeneity of the tumor microenvironment and the differing energetic demands of stem-like versus differentiated tumor cells.

### 2.4. Correlation Analysis Between the APC Signature Genes and Metabolic Pathways

**Figure 4:**
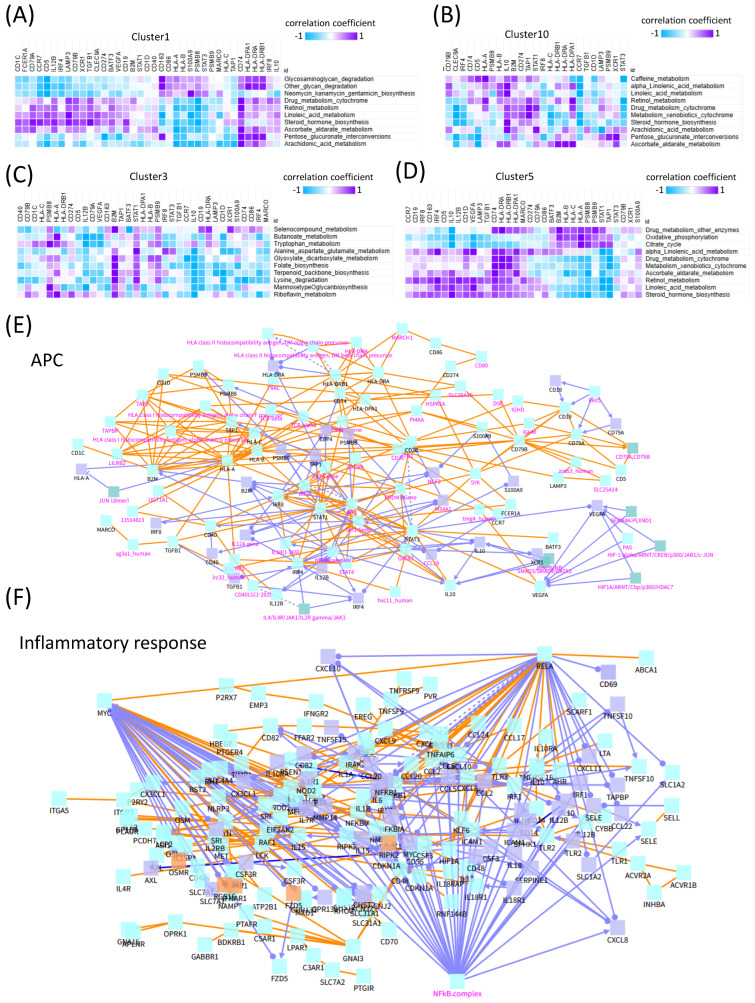
Correlation analysis between the antigen-presenting-cell (APC) signature genes and metabolic pathways. (**A**) A heatmap showing the correlations between APC genes and highly enriched metabolic pathways in cluster 1. (**B**) A heatmap showing the correlations in cluster 10. (**C**) A heatmap showing the correlations in cluster 3. (**D**) A heatmap showing the correlations in cluster 5. (**E**,**F**) Functional interaction networks of immune signaling and inflammatory response based on curated pathway data. Network graphs illustrate key molecular interactions involved in (**E**) antigen presentation and immune recognition and (**F**) inflammatory signaling. Nodes represent genes, proteins, or protein complexes, while edges indicate known biological relationships. Edge colors reflect interaction types: Orange: protein–protein interactions, Purple: gene regulatory interactions, Node label colors denote their roles in the network: Black labels mark seed nodes (user-supplied genes of interest) Magenta labels indicate intermediate nodes inferred through network expansion algorithms. These networks highlight critical signaling hubs (e.g., HLA complex in (**E**) and NF-κB complex in (**F**)) and their connections to peripheral components, offering insight into how immune and inflammatory pathways are coordinated at the molecular level. Protein–protein-interaction (PPI) networks were generated using ConsensusPathDB, incorporating only the experimentally validated functional interactions from curated databases (e.g., BioGRID, IntAct, and Reactome). A confidence score threshold of 0.7 was applied to include only the high-confidence interactions. Hub genes such as MYC, RELA, and NFKB1 were identified based on network connectivity (node degree).

To further investigate which APC-related genes were associated with metabolic activity, we analyzed the correlations between APC gene expression and metabolic pathways. Among the ten metabolic pathways highly active in low-stemness clusters, clusters 1 and 10 exhibited distinct correlation patterns. In cluster 1, *CD74* showed positive correlations with nine of the ten metabolic pathways. In cluster 10, *IL10* was positively correlated with nine metabolic pathways. In the high-stemness cluster 3, *B2M* was positively associated with eight metabolic pathways. Similarly, in the high-stemness cluster 5, five APC-related genes—*CD74*, *HLA***-***DRA*, *HLA-DRB1*, *HLA-DPA1*, and *MARCO*—were positively correlated with eight metabolic pathways (Figure 4A,B).

These results suggest that low-stemness, low-entropy cells may contribute to the formation of an inflammatory tumor microenvironment while evading immune surveillance through APC gene expression. This phenomenon was particularly evident in the mesenchymal-like differentiated states, characterized by low stemness and heightened TNF-α signaling and inflammatory responses (Figure 4C,D).

In GBM, cells with low stemness often display the activation of inflammatory pathways, including TNF-α and NF-κB signaling. This inflammatory profile is typically associated with more differentiated cellular states, such as the mesenchymal-like phenotype. In contrast, GSCs tend to exhibit immune-evasive characteristics and suppress inflammatory responses. These observations suggest that increased inflammatory signaling is inversely correlated with stemness, highlighting the dynamic heterogeneity and state transitions in GBM (Figure 4C,D).

To further elucidate these relationships, we constructed protein–protein-interaction (PPI) and gene-regulatory networks incorporating APC genes and inflammatory response signature genes. A network analysis identified *MYC*, *RELA*, and *NFKB1* as the central hub proteins, indicating their pivotal roles in regulating these pathways (Figure 4E,F).

## 3. Discussion

In this study, we identified a novel association between elevated APC signature gene expression [11] and reduced stemness, coupled with metabolic reprogramming, in GBM. While APCs are traditionally understood as key players in immune surveillance and T cell activation, our findings suggest that their transcriptional signatures also reflect intrinsic states of GBM cells, particularly regarding stemness and metabolism [12].

A key observation was the inverse correlation between APC signature genes, including *HLA-DRA*, *CD74*, *CD80*, *CD86*, and *CIITA*, and GSC markers. This suggests that elevated APC-associated gene expression corresponds to more differentiated, less stem-like tumor-cell states. These findings aligned with recent studies showing that GBM stemness is regulated not only by intrinsic signaling pathways (e.g., Wnt/β-catenin and Notch) but also through immune evasion and metabolic interactions within the tumor microenvironment. Thus, APC-high GBM states may be associated with a more immunologically active and transcriptionally restricted cellular state. Our findings suggest that APC gene expression patterns may serve as transcriptional indicators of distinct GBM cell states, particularly in relation to stemness and metabolic activity, rather than acting as direct effectors of these phenotypes.

Interestingly, the APC-enriched states also exhibited the upregulation of the inflammatory response and TNF-α signaling pathways. This contrasted with the canonical view that inflammation broadly promotes tumor progression and stemness, especially in immune-privileged sites such as the brain. Instead, our data suggest that inflammatory signals in GBM may act in a context-dependent manner—for example, promoting differentiation in mesenchymal-like or low-stemness tumor states while supporting proliferation or immune evasion in proneural or high-stemness contexts—thereby potentially enhancing or suppressing tumor immunogenicity depending on cellular state.

This interpretation is supported by previous studies showing that TNF-α signaling can reduce stemness by promoting differentiation or senescence-like states in glioma and other cancers. For example, TNF-α/NF-κB activation has been shown to induce astrocytic differentiation and suppress glioma stem-like cell phenotypes [13,14], as well as trigger senescence-associated transcriptional programs under inflammatory conditions [15]. These findings suggest that inflammatory signaling may, in specific contexts, oppose self-renewal and favor cellular maturation.

From a metabolic perspective, the APC-high GBM states showed a shift from glycolytic metabolism toward increased oxidative phosphorylation and lipid metabolism. This supports the hypothesis that immune-instructed differentiation is coupled with metabolic remodeling, which reduces the bioenergetic flexibility of GSCs and renders these cells more vulnerable to environmental stress and therapeutic interventions. Such metabolic transitions may also make tumors more susceptible to inhibitors targeting lipid oxidation or mitochondrial metabolism, especially in patients with high APC gene expression [16,17].

Importantly, these findings have potential clinical implications. The co-enrichment of APC signatures with metabolic and inflammatory programs could serve as a composite biomarker to stratify patients with GBM according to tumor differentiation and immune engagement status. These patient subgroups might be more responsive to immunotherapies or metabolic-targeted treatments. Furthermore, APC-related gene expression profiles could enhance our understanding of mechanisms underlying immunotherapy resistance, particularly in GBMs marked by immunosuppression and GSC dominance [18]. Given its association with both tumor cell differentiation and immune pathway activation, the APC metabolic signature may serve as a prognostic indicator of tumor aggressiveness and as a potential predictive biomarker of response to immunotherapy or metabolic-targeted interventions.

Nonetheless, this study had limitations. Our analyses relied primarily on transcriptomic datasets and inferred activity scores, which do not fully capture functional APC-T cell interactions or spatial organization within the TME. Future investigations incorporating spatial transcriptomics and in situ proteomics will be crucial to dissect how APC activity directly modulates GSC niches and tumor progression. A major limitation of this study was its exclusive reliance on transcriptomic inference; we did not perform functional validation using patient-derived tissue (e.g., IHC or spatial proteomics) or experimental models, which will be critical for confirming causal relationships and translational relevance.

Additionally, experimental validation using in vitro and in vivo models is necessary to establish causality in the observed associations.

In conclusion, we propose that APC signature gene expression is not simply a marker of immune cell infiltration but reflects a broader immuno-metabolic program that influences GBM cell identity. Decoding these signatures enhances our understanding of GBM heterogeneity and reveals new vulnerabilities for therapeutic targeting [19].

## 4. Materials and Methods

### 4.1. Bulk RNA-Seq Analysis

We utilized RNA sequencing (RNA-seq) data for GBM from The Cancer Genome Atlas (TCGA) (https://portal.gdc.cancer.gov/projects/TCGA-GBM, accessed on 1 May 2025) for our analysis. Survival analysis was performed using GEPIA2 [20], and gene ontology analysis was conducted using Metascape [21]. We analyzed bulk RNA sequencing data from the TCGA-GBM cohort, which included transcriptomic profiles from 152 patients.

For the TCGA-GBM bulk RNA-seq data, we used the upper quartile normalization and log2 transformation of TPM values. Samples with >20% missing values or low-quality metrics were excluded. Batch effect correction across the TCGA batches was performed using the Combat function from the sva R package, version 4.0.3.

Deconvolution analysis was performed using the xCell algorithm [22] to estimate the enrichment scores of various immune and stromal cell types from the bulk RNA-seq data. This approach was previously applied in a similar context in our prior work [9].

Protein–protein interaction and gene regulatory network analyses were performed using ConsensusPathDB (CPDB) [23]. Gene set variation analysis (GSVA) was carried out using the *GSVA* R package [24].

### 4.2. Single Cell RNA-Seq Analysis

The single-cell RNA sequencing (scRNA-seq) data for GBM were obtained from GSE131928 (https://www.ncbi.nlm.nih.gov/geo/query/acc.cgi?acc=GSE131928, accessed on 1 May 2025).

For the single-cell RNA sequencing analysis, we utilized the GSE131928 dataset, comprising approximately 8000 cells from 5 adult GBM patients and 5000 cells from 3 pediatric GBM patients. For the scRNA-seq analysis, we used the Scanpy version 1.11.2. Gene expression matrices were log-normalized using the pp.normalize_total and pp.log1p functions (Scanpy version 1.11.2). Highly variable genes were selected with pp.highly_variable_genes, and cells with fewer than 200 genes or >10% mitochondrial gene content were excluded. Batch correction was performed using Harmony 1.2.3 for the integration of the adult and pediatric datasets. Clustering was performed with the Leiden algorithm, and dimensionality reduction was performed with UMAP.

Stem-like cells at the single-cell level were identified using the StemID tool (StemID2 is available in RaceID version 0.3.9) [25]. StemID was used to calculate the transcriptional entropy scores for each cell, with higher entropy indicating higher stemness potential. Clustering was performed using the Leiden algorithm (Version: 0.4.3.1), and the clusters were ranked based on their mean entropy scores. High-entropy clusters were classified as high-stemness states and validated by the elevated expression of canonical glioma stem-cell markers such as SOX2, OLIG2, and PROM1. Low-entropy clusters were enriched for APC-related and inflammatory gene expression.

APC signature genes and cancer hallmark pathways were retrieved from the Molecular Signatures Database (MSigDB) [26]. Metabolic reprogramming was analyzed using 83 KEGG-defined metabolic pathways [27]. We applied the Shapiro–Wilk test for normality. Gaussian distributions were analyzed using the unpaired, two-tailed Student’s t-test, and non-Gaussian distributions were analyzed using the Mann–Whitney U test. Multiple comparisons were corrected via Bonferroni or Benjamini–Hochberg FDR methods, where appropriate.

Correlations between APC gene expression and metabolic pathway activity scores were computed using a Pearson correlation analysis. For each APC gene–pathway pair, Pearson correlation coefficients (r) and corresponding *p*-values were calculated using the scipy.stats.pearsonr function in Python. To account for multiple testing, *p*-values were adjusted using the Benjamini–Hochberg false discovery rate (FDR) correction method. Only associations with FDR-adjusted *p*-values of <0.05 were considered statistically significant and visualized in the heatmaps.

### 4.3. Network Analysis

Protein–protein interaction (PPI) and gene regulatory networks were constructed using the ConsensusPathDB (CPDB) database, which integrates curated functional interaction data from multiple sources (e.g., BioGRID, IntAct, and Reactome). Only experimentally validated physical or biochemical interactions were considered. A minimum interaction confidence score threshold of 0.7 (high-confidence interactions) was applied to filter the networks. Nodes such as MYC, RELA, and NFKB1 emerged as central hubs based on their degree of connectivity within the network.

### 4.4. Statistical Analysis

For all comparisons involving continuous variables, we first assessed the normality of the data distribution using the Shapiro–Wilk test. If the data met the assumption of normality, we used the unpaired two-tailed Student’s t-test to compare group means. In cases where normality was not assumed, we applied the Mann–Whitney U test for non-parametric comparisons. All statistical analyses were performed using Python (SciPy v1.10) or R (v4.2), and *p*-values were adjusted using the Benjamini–Hochberg false discovery rate (FDR) method, where applicable.

## Data Availability

No new data were created or analyzed in this study.
